# Insight into the Interaction of Metal Ions with TroA from *Streptococcus suis*

**DOI:** 10.1371/journal.pone.0019510

**Published:** 2011-05-18

**Authors:** Beiwen Zheng, Qiangmin Zhang, Jia Gao, Huiming Han, Ming Li, Jingren Zhang, Jianxun Qi, Jinghua Yan, George F. Gao

**Affiliations:** 1 CAS Key Laboratory of Pathogenic Microbiology and Immunology, Institute of Microbiology, Chinese Academy of Sciences, Beijing, China; 2 College of Life Sciences, Graduate University, Chinese Academy of Sciences, Beijing, China; 3 The Key Laboratory of Ministry of Education for Microbial and Plant Genetic Engineering, Guangxi University, Nanning, Guangxi, China; 4 School of Medicine, Tsinghua University, Beijing, China; 5 Beijing Institutes of Life Science, Chinese Academy of Sciences, Beijing, China; University of Queensland, Australia

## Abstract

**Background:**

The scavenging ability of sufficient divalent metal ions is pivotal for pathogenic bacteria to survive in the host. ATP-binding cassette (ABC)-type metal transporters provide a considerable amount of different transition metals for bacterial growth. TroA is a substrate binding protein for uptake of multiple metal ions. However, the function and structure of the TroA homologue from the epidemic *Streptococcus suis* isolates (SsTroA) have not been characterized.

**Methodology/Principal Findings:**

Here we determined the crystal structure of SsTroA from a highly pathogenic streptococcal toxic shock syndrome (STSS)-causing *Streptococcus suis* in complex with zinc. Inductively coupled plasma mass spectrometry (ICP-MS) analysis revealed that apo-SsTroA binds Zn^2+^ and Mn^2+^. Both metals bind to SsTroA with nanomolar affinity and stabilize the protein against thermal unfolding. Zn^2+^ and Mn^2+^ induce distinct conformational changes in SsTroA compared with the apo form as confirmed by both circular dichroism (CD) and nuclear magnetic resonance (NMR) spectra. NMR data also revealed that Zn^2+^/Mn^2+^ bind to SsTroA in either the same site or an adjacent region. Finally, we found that the folding of the metal-bound protein is more compact than the corresponding apoprotein.

**Conclusions/Significance:**

Our findings reveal a mechanism for uptake of metal ions in *S. suis* and this mechanism provides a reasonable explanation as to how SsTroA operates in metal transport.

## Introduction

*Streptococcus suis* serotype 2 (*S. suis* 2) is a gram-positive coccus that causes diseases in pigs and humans, and is therefore a zoonotic pathogen [1], [2]. Since the first human case of *S. suis* 2 infection was described in Denmark in 1968, over 400 human *S. suis* infection cases have been recorded thus far, covering nearly 30 countries [2], [3]. In 1998 and 2005, *S. suis* 2 human infections, with the new disease form of streptococcal toxic shock syndrome (STSS), had been reported in China, following the outbreaks in swine stocks initially, which caused a total of 240 human infections and claimed 52 lives [4]. Our group, in collaboration with other groups, has sequenced the entire genomes of the representatives of these two virulent *S. suis* 2 isolates (05ZYH33 and 98HAH12) in 2007 [5] and laid the foundation for the study of the STSS-causing *S. suis* 2 at the molecular level to reveal its pathogenicity [1], [6], [7].

Almost one third of proteins in nature depend on a particular metal for their diverse functions [8] and divalent metal cations are essential for bacteria [9]. For instance, manganese plays a primary antioxidant role in bacteria [10] and affects bacterial pathogenesis [11]. Zinc is one of the most abundant metals in bacteria and is an essential co-factor of many metabolic enzymes and transcription factors [12]. The Mn^2+^ ion is characterized as hard metal and tends to prefer hard ligands, while zinc prefers soft ligands [13]. To obtain appropriate cellular concentrations of transition metal ions, bacteria have evolved elaborate machineries (such as ABC-transporters and ion channels) to transport these ions across biological membranes.

ABC transporters and metal ions are significant for bacteria growth and virulence in *Streptococcus* [14]. However, only recently have insights emerged into the metal metabolism of *S. suis*. The *adcR* gene (which encodes the regulator of the Adc operon) was isolated by divalent cation deprivation [15]. Furthermore, the extracted cell surface proteins of *S. suis* from mutants defective in genes regulating metal ion uptake are able to confer significant protection against *S. suis* 2 infection in mice [16]. Previously, we identified a global zinc-response regulator of the Zur family (zinc uptake regulator) from *S. suis* 2 [17], unveiling its relationship with zinc homeostasis in this organism. Whole-genome sequence analysis led to the proposition that *S. suis* expresses two putative transition metal transport systems, encoded by the Adc operon [15] and Tro operon (Table S1), consistent with the situation in many other bacterial pathogens that harbor several metal transporters.

**Figure 1:**
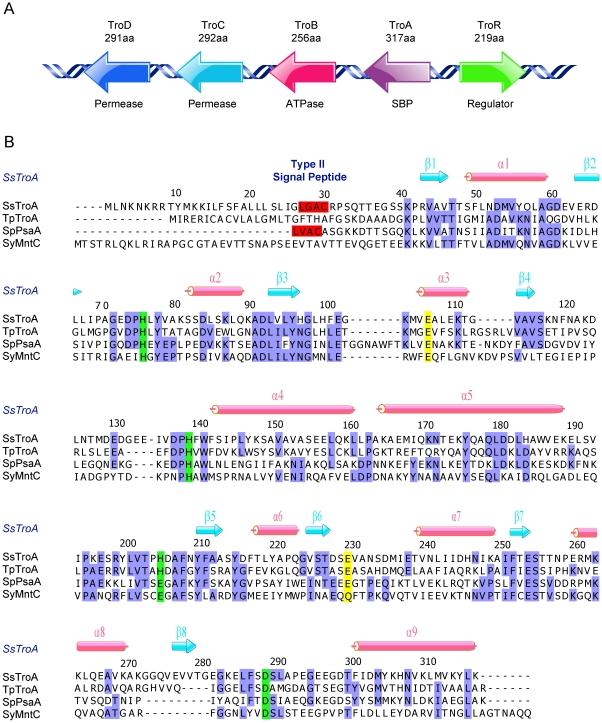
Genetic organization of the *S. suis* Tro operon and alignment of SsTroA homologues. (A) Gene descriptions of the *S. suis* Tro operon. (B) Structure-based multiple sequence alignment of SsTroA homologues. The most-conserved residues in all homologues are shaded blue. The four metal-coordinating residues are highlighted in green. The LXXC lipoprotein motif (type II signal peptides for Sec-dependent transport) [34] is highlighted in red. The two conserved residues predicted to form the salt bridge are marked in yellow. Secondary structure elements for SsTroA are shown above the sequence. TpTroA, TroA from *Treponema pallidum*; SpPsaA, PsaA from *Streptococcus pneumonia*; SyMntC, MntC from *Synechocytis* 6803.

TroA was isolated from outer membrane preparations of *Treponema pallidum* (TpTroA) [18]. This protein belongs to a well-studied family, formerly designated as cluster 9 substrate binding protein (SBP) [19], but recently reclassified into the A-1 family of prokaryotic SBPs [20]. The SsTro operon contains five genes, an ATP-binding cassette metal transport system (*troABCD*) (encoding four proteins) and a transcriptional regulator (*troR*) (Fig. 1A). The crystal structures of some A-1 family SBPs have been determined over the past decade. These structures include Mn-specific SBPs (e.g.,TpTroA [21]–[22], *Streptococcus pneumoniae* PsaA [23], and *Synechocystis* 6803 MntC [24]), Zn-specific SBPs (e.g., *S. pneumoniae* AdcAII [25], *Synechocystis* 6803 ZnuA [26], [27], and *S. pyogenes* Lbp [28]) and Fe-specific SBPs (*Streptococcus pyogenes* MtsA [29]). They share a common structure of two (α/β)_4_ domains linked by a long helix, with the metal bound at the interface between the domains. Hazlett *et al.* [30] and Desrosiers *et al.* [31] revealed that TpTroA has high binding affinity for both Zn^2+^ and Mn^2+^, but Zn^2+^ is considered to be the primary substrate of TpTroA [32].

Although the A-1 family SBPs has been extensively characterized they have been the subject of considerable controversy with respect to their metal binding properties. When bound to metal, by CD spectra TpTroA does not undergo structural changes, however, *T. pallidum* ZnuA undergoes conformational changes when Zn^2+^ is loaded [31]. *Synechocystis* 6803 ZnuA displays concomitantly large conformational changes in two of its three chelating histidines due to the release of Zn because it possesses two Zn binding sites [27]. However, the second binding site is absent in other transporters (e.g., TpTroA), and therefore its exact biological function remains unclear [33]. To obtain further insight into the function of SsTroA, we determined the X-ray crystal structure of the Zn^2+^-bound form of the SsTroA at a resolution of 2.6 Å, and further characterized the recombinant SsTroA. Binding of divalent metal cations to SsTroA, induces a substantial conformational reorganization to a more ordered state. Our structural and functional data reported here lay out the scenario of SsTroA participating in *S. suis* divalent metal homeostasis.

## Results

### *In silico* analyses, cloning, purification and biochemical characterization of SsTroA

*In silico* analysis revealed that SsTroA is a putative lipoprotein (Fig. 1B). The amino-terminal signal sequence of such lipoproteins is distinguishable by the presence of a lipobox motif at the signal sequence cleavage site. The conserved lipobox motif is usually characterized as L_−3_-[A/S/T]_−2_-[G/A]_−1_-C_+1_ and lipid modification is achieved through a covalent linkage of a diacylglyceride to the conserved cysteine residue. Indeed, the cysteine residue is absolutely conserved in all bacterial lipoproteins [34], [35]. SsTroA contains a typical type II signal peptide for Sec-dependent transport. Its presumptive lipobox sequence is LGAC, containing the indispensible cysteine in position 30, indicating that SsTroA is not released into the environment but rather inserted into the plasma membrane via a diacylglyceryl anchor. Alignment of sequences also revealed that the metal binding site of SsTroA is very similar to Mn-specific SBPs (Fig. 1B). These data suggested that the SsTroA should physiologically bind Mn^2+^.

We cloned the mature SsTroA domain (residues 36–317), and the protein was purified using GST-affinity column and a subsequent gel-filtration chromatography (Fig. S1A). Samples collected from elution fractions were separated by 12% SDS-PAGE (Fig. S1A) and the results show that SsTroA has a molecular mass of ∼34 kDa, in consistence with the theoretical monomer molar mass of 32 kDa. An analytical FPLC Superdex® 200 profile revealed that SsTroA exists as a mixture of monomer and dimer in solution, though mainly as a monomer (Fig. S2A). To determine the native mass of SsTroA (unaffected by protein shape), quantitative hydrodynamic analyses were performed by analytical ultracentrifugation. Analysis of the sedimentation velocity profiles using the SEDFIT program yielded the weight average molar mass of the major peak is 31.8 kDa (Figure S2B), which accounts for 90% of the total protein (i.e., nearly identical to the theoretical molar mass of 32 kDa). Another peak at 62.6 kDa (6.8% of the total) is believed to correspond to dimers. The other ∼3.2% of the protein has a significantly-higher sedimentation coefficient, but the amount of this subpopulation did not change significantly as a function of protein concentration. Thus, our data suggest that under the solution conditions used, a small portion of SsTroA is involved in the formation of stable complexes. This is consistent with the evidence that periplasmic SBPs exist in a monomer/dimer equilibrium, with monomers having higher affinity for the substrate [36], [37].

To determine if SsTroA is expressed in *S. suis* 2, total cellular proteins from *S. suis* 2 cells, purified SsTroA, and negative control protein (Ss1661 protein from *S. suis* 2) were separated by SDS-PAGE, transferred to a nitrocellulose membrane (GE Healthcare), and probed with anti-SsTroA serum. A 34-kDa protein band was detected in the *S. suis* 2 cell lysate (Fig. S1B), indicated that SsTroA is expressed in the bacteria.

### Metal binding stoichiometries and binding affinities

To investigate the presence of divalent metal ions in the purified SsTroA and assess the ability of SsTroA to bind metal ions, the recombinant protein was subjected to a test of inductively coupled plasma mass spectrometry (ICP-MS) analysis. The metal contents of SsTroA as isolated or after reconstitution were shown in Table 1. The stoichiometry was found to be 0.96±0.2 Zn^2+^/SsTroA monomer and 0.89±0.2 Mn^2+^/SsTroA monomer, which suggests that recombinant SsTroA protein binds Zn^2+^ and Mn^2+^ roughly in a 1∶1 ratio. The ICP-MS data provided support for one metal binding site per SsTroA monomer, as observed in TpTroA and *Neisseria gonorrhoeae* MntC [31], [33]. This led us to evaluate the metal interactions with the SsTroA protein in more details.

**Table 1:** Metal contents of SsTroA after initial isolation or after reconstitution.

Metal ions	Metal in SsTroA^a^
After initial isolation	
Zn^2+^	0.8±0.02 (n = 3)
Mn^2+^	0.06±0.03 (n = 3)
After reconstitution	
Zn^2+^	0.96±0.2 (n = 3)
Mn^2+^	0.89±0.2 (n = 3)

a Mean ± the standard error of the mean.

**Figure 2:**
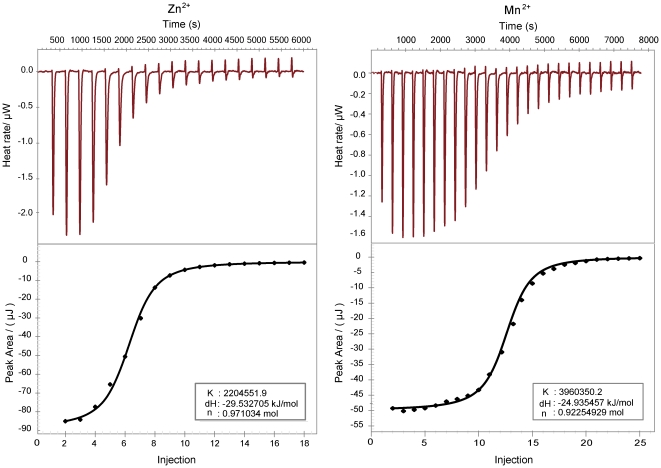
ITC analysis of the SsTroA interaction with Zn and Mn. Left: apo-SsTroA (90 µM) with addition of Zn^2+^ (500 µM); Right: apo-SsTroA (30 µM) with Mn^2+^ (200 µM). In each case, the upper panel shows raw energy changes during the titration, while the lower panel presents the integrated peak areas. The fitting of the data yielded the thermodynamic parameters listed in Table S2.

We applied isothermal titration calorimetry (ITC) to monitor the energetics of Zn^2+^ and Mn^2+^ binding to the apo-SsTroA. ITC measures the heat directly released or absorbed upon an interaction triggered by mixing two components, and is capable of calculating both the extent of the ligand binding affinity and the free energy values (ΔG) and enthalpy(ΔH) changes, from which entropy (ΔS) is determined [38]. The isotherms for loading with Zn^2+^ and Mn^2+^ appeared in normal sigmoidal titration curves, in agreement with our ICP-MS data. Again the binding stoichiometry (*n*) was determined to be ∼1 in both cases (Fig. 2), confirming that a specific 1∶1 complex is formed in each case. The association constants (*Ka*) for each metal ion binding to SsTroA were around 10^7^ M^−1^ (Fig. 2). SsTroA affinities for Zn^2+^ and Mn^2+^ were calculated from duplicate measurements with K_D_s ( = 1/Ka) of 434±9 nM and 254±13 nM, respectively, indicating the formation of tight complexes. It should be noted that SsTroA binds Mn^2+^ with a higher affinity than that of Zn^2+^. These values are of a similar magnitude to those described for binding of Zn^2+^ and Mn^2+^ to *N. gonorrhoeae* MntC [33]. In each metal ion titration with both metal ions, we observed negative enthalpy and entropy values, demonstrating that binding of each ion to SsTroA is an exothermic event. The thermodynamic profiles for metal binding by SsTroA are not identical to those previously reported for TpTroA [31]. Intrinsic metal binding to proteins is usually entropically driven (Δ*H*>0) due to the very high dehydration energies of divalent cations. However, the binding enthalpy can become exothermic (Δ*H<*0) if metal binding is coupled to a protein conformational change [39], [40]. Our ITC data are consistent with a significant conformational change observed in our nuclear magnetic resonance (NMR) and CD spectra. Thermodynamic parameters for the binding of Zn^2+^/Mn^2+^ to SsTroA are summarized in Table S2.

### Room temperature Electron Paramagnetic Resonance (EPR) monitoring of Mn^2+^-SsTroA complex

**Figure 3:**
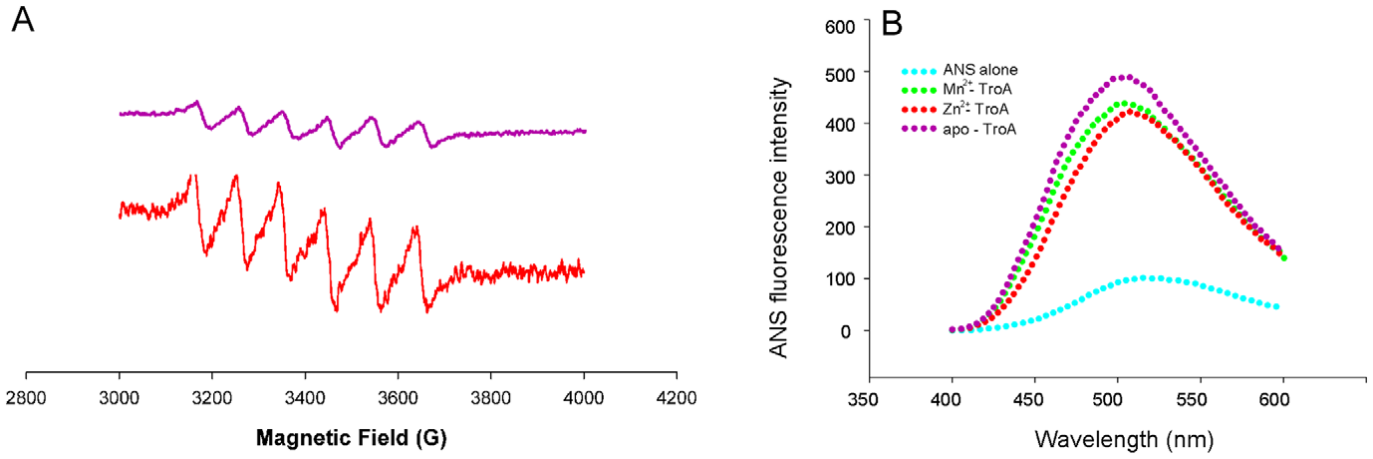
X-band EPR spectra and ANS fluorescence analysis of SsTroA. (A) EPR spectra of Mn^2+^ (100 µM) in the absence (red line) and presence (purple line) of SsTroA (100 µM) at room temperature in solution. The total Mn^2+^ concentration was the same in both samples. The signal intensity decrease corresponds to the zero-field splitting arising from the disturbances in the octahedral ligand field of the bound metal. The instrument conditions are as follows: microwave power, 20 mW; microwave frequency, 9.53 GHz; modulation frequency, 100 KHz; modulation amplitude, 1 G; and modulation amplitude, 1.0 mT. (B) Fluorescence changes in the intensity emission for SsTroA indicate a metal dependent decrease in hydrophobic residue exposure. Emission spectra of ANS in the presence of different SsTroA states: apo-SsTroA (purple), Zn^2+^-SsTroA (green) and Mn^2+^-SsTroA (red). A significant enhancement in fluorescence is probed on binding of ANS to apo-SsTroA. This fluorescence is slightly diminished in the presence of metal ions, which indicates the ordering of the metal binding domain of the SsTroA structure when metal is bound.

EPR spectroscopy is a useful tool that allows one to probe differences in the metal centers of proteins in solution that may not be apparent in the crystal structures [41]. To further investigate the SsTroA metal center, sample of 100 µM apo-SsTroA was loaded with 100 µM MnCl_2_. The X-band EPR spectra of Mn^2+^-SsTroA at room temperature displays a typical six-line pattern (Fig. 3A). High-spin Mn^2+^ centers typically exhibit EPR resonances with characteristic six-line splitting patterns due to hyperfine interactions between the unpaired electrons and the *I = *5/2 ^55^Mn nucleus, indicating the binding of the Mn^2+^ to the protein [42]. Upon entrance of the Mn^2+^ into the binding site, the Mn^2+^ bound protein reduces the coordination of weakfield water, increasing spin-orbit interactions and zero-field energies relative to that of Mn(H_2_O)_6_^2+^. As observed, both titration traces had similar spectra but with varying intensities of the signal. These data clearly illustrate that high-affinity Mn^2+^ binding by SsTroA makes SsTroA sensitive to scavenging low levels of free Mn ions in the surrounding environment.

### Decreased fluorescence intensity of 1-anilino naphthalene-8-sulfonic acid (ANS) with SsTroA upon metal loading

To examine whether ligand binding induces a variation of exposed hydrophobic protein regions, we applied fluorescence spectroscopy to monitor the binding of the hydrophobic probe ANS to SsTroA. ANS is extensively used to detecting the equilibrium and kinetic folding intermediates of proteins, especially for a partially folded protein [43]. Interaction of the probe with a hydrophobic site yields an enhancement of fluorescence intensity, usually accompanied by a blue shift of the maximum. SsTroA significantly enhances the emission intensity of ANS and promotes a blue shift (Fig. 3B). Meanwhile, the fluorescence intensity of ANS clearly decreases in the metal bound form compared to the apo-SsTroA. As indicated by the diminished fluorescence, ANS displays stronger binding to Mn^2+^-SsTroA than Zn^2+^ -SsTroA. The spectra clearly provide evidence that the addition of metal ions induces a large change in the nonpolarity of the ANS-binding site, and the Mn^2+^ causes a greater change than Zn^2+^ does. These data suggested that the decreased plots observed in the spectra (Fig. 3B) can be attributed to a more compact and ordered structure for SsTroA in the presence of metal.

### Conformational changes and enhancement of thermal stability

**Figure 4:**
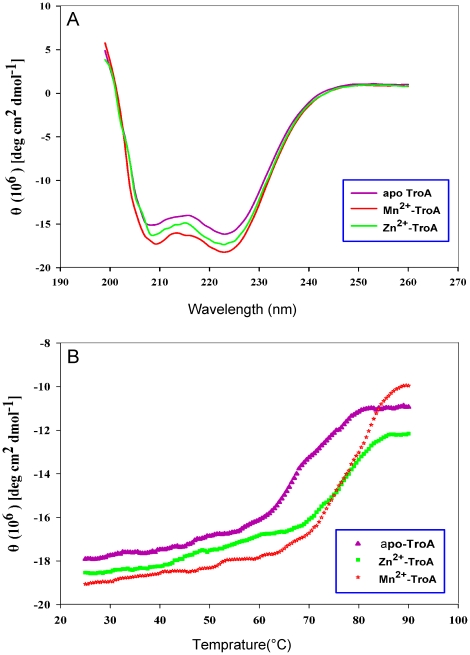
CD spectra of SsTroA. (A) Metal ions induced conformational changes monitored by far-UV CD spectra. Far-UV CD spectra were acquired for apo-SsTroA (purple) and SsTroA in the presence of 100 µM Zn^2+^ (green), or 100 µM Mn^2+^ (red). (B) Thermal unfolding followed by far-UV CD spectra at 223 nm. Data for apo-SsTroA (purple) and SsTroA in the presence of 100 µM Zn^2+^ (green) or 100 µM Mn^2+^ (red).

As protein aggregation occurs after demetallization, we suspected that apo-SsTroA is unstable compared to the native SsTroA at room temperature, i.e., we assumed that SsTroA displays metal-dependent conformational stability of SsTroA. To obtain further information on the metal sensitivity of the SsTroA and details on possible structural changes occurring upon its interaction with metal ions, we applied CD spectroscopy to monitor these processes. Proteins were prepared by incubation with Zn^2+^ and Mn^2+^ as previously described [31]. In the far-UV regions (Fig. 4A), the CD spectra are generally characterized by distinct minima at 208 and 223 nm respectively, which are the features of proteins that contain α-helix conformational elements, consistent with the crystal structure data. The metal bound SsTroA samples both displayed spectra similar to apo-SsTroA, only with minor differences, though both has deeper 208 nm and 223 nm minima relative to apo-SsTroA. The intensity of the 223-nm band of SsTroA is in the order: apo-SsTroA>Zn^2+^-SsTroA>Mn^2+^-SsTroA, suggesting a decrease of the content of secondary structure in the apo form compared to the metal-bound forms. While previous work demonstrates that TpTroA does not manifest changes discernible by CD upon metal binding, in contrast, *T. pallidum* ZnuA does undergo conformational changes when Zn^2+^ is loaded [31]. These data indicated that TpTroA and SsTroA present differential binding behaviors upon metal binding.

Thermal denaturation transitions were assessed by monitoring the temperature dependent change in ellipticity at 223 nm to determine apparent thermal denaturation midpoints in the presence or absence of metal ions. As expected, thermal denaturation proceeded, as expected, with a sharp decrease in ellipticity at 223 nm at the melting temperature and a complete loss of secondary structure (Fig. 4B). The data are fit very well to a two-state model between the folded and unfolded states. Nonetheless, the unfolding profiles are obviously different, the heat denaturation analysis revealed that the apoprotein begins to dramatically lose CD signal at 60–65°C, whereas the Zn-bound or Mn-bound protein starts to significantly lose CD signal at a temperature as high as 65–70°C. The apoprotein exhibits a half-denaturation (*T_m_*) of 71.5±0.1°C, and addition of Zn^2+^ and Mn^2+^ significantly stabilizes SsTroA as judged by a substantial enhancement in the *Tm* value (Δ*Tm* = 4.6±0.2°C and 5±0.1°C, respectively), which agrees with the presence of Zn^2+^ or Mn^2+^ as seen in CD spectroscopic changes (Fig. 4A). These results combined with CD spectrum data imply that the ternary protein-metal complex appears to form a compact domain organization, i.e., the metal-ligand interactions in the SsTroA protein indeed play an important role in imparting extra stability to the metal binding site of the protein. Further, as we proposed that Mn^2+^ has a more significant effect on the conformation and thermal stability of SsTroA than Zn^2+^.

### Global features and geometry of metal binding

**Figure 5:**
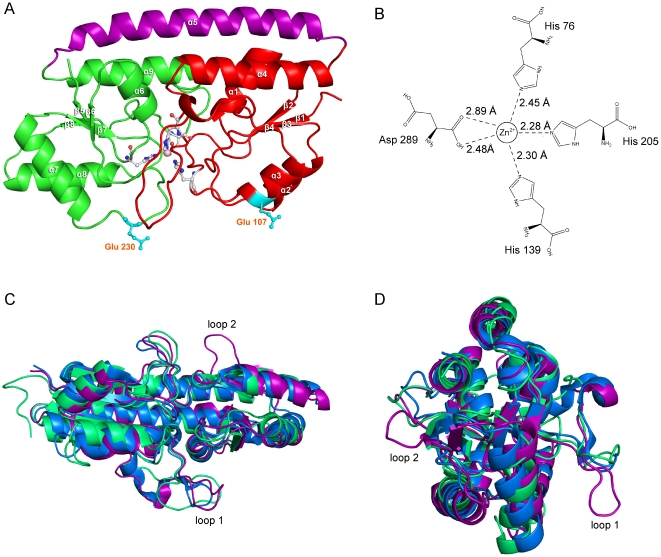
Crystallographic structure of SsTroA and structural comparisons of SsTroA with structurally known Mn-specific SBPs. (A) Cartoon diagram of the SsTroA, the N-terminal domain is shown as red, the C-terminal domain is green, and the linking helix is colored purple. The residues forming the metal binding site are presented in sticks-balls format, two conserved Glu residues predicted to form the salt bridge are displayed in cyan, and the zinc ion is displayed as a lightpink sphere. The α-helices are designated α1–α9, and the β strands are β1–β8. (B) The coordination bonds formed between the Zn^2+^ and His 76, His 139, His 205 and Asp 289. The distances between Zn^2+^ and these residues are calculated. (C) Bottom view of the structural comparison. (D) Side view of the comparison. Comparisons shown here are SsTroA (purple), TpTroA (marine), and *S. pneumoniae* PsaA (limegreen). The unique loops are designated as Loop 1 and Loop 2. Structures are displayed in ribbon representation.

To gain structural insight into SsTroA function, we determined the crystal structure of Zn-bound SsTroA. The X-ray crystal structure was solved by molecular replacement using TpTroA as the search model (PDB code: 1K0F) [22]. The final structure was refined to a resolution of 2.6 Å and consists of 278 amino acids (residues 40–317). Phasing and refinement statistics are reported in Table S3. The overall structure of SsTroA is typical of SBPs in the A-1 family [19]. The peptide chain crosses between the N-terminal and C-terminal domains only once linking the two (β/α)_4_ domains via a 33-residue kinked rigid α-helical backbone that runs the full length of the molecule (Fig. 5A) and the metal binding site is buried in the interdomain interface. The N-terminal domain (residues 40–164) is a β/α sandwich-like domain, consisting of a four-stranded β-sheet with 2-1-3-4 linking topology, and with an α-helix connecting each strand. The C-terminal domain (residues 196–317) is also a β/α sandwich composed of a four stranded parallel β-sheet with 2-1-3-4 linking topology, with both sheets sandwiched between layers of α-helices. The two independent domains interact with each other across a large interface that creates the binding pocket for the metal ligand.

In our structure, we clearly observed the metal binding pocket provided by the buried surface between the N-terminal and C-terminal domains (Fig. 5B). Independent experimental evidence has demonstrated that the ion bound in the metal binding pocket of recombinant SsTroA is Zn^2+^ (see metal content analysis). Zinc is pentacoordinated by His 76 (bond length, 2.14 Å), His 139 (2.11 Å), His 205 (2.18 Å) and Asp 289 (2.20 Å, 2.38 Å). His 76 and His 139 are provided by the N-terminal domain, while His 205 and Asp 289 are contributed by the C-terminal domain. The Zn coordination in our structure is best described as distorted square pyramidal [21]. The N^ε2^ nitrogen atoms of His 76 and His 139, with the O^ε1^ an O^ε2^ atoms of Asp 289 generated the square planar. While the N^ε2^ atom of His 205 is the summit of the square pyramid. The binding site is buried ∼7 Å below the molecule surface and the four coordinating residues are also completely buried. This coordination geometry and the amino acid types are very similar among the Mn-binding SBP family [21], [23], [24].

Despite the determination of several SBP structures, the structural difference between Zn-specific and Mn-specific proteins remain unclear [44]. There exists difference with SBPs, specific either for zinc or manganese. In the Zn-specific ZnuAs, the metal is coordinated by 3 histidines plus a water or glutamate [44], [45]. In the case of Zn-specific AdcAII, the residues involved in Zn binding are three histidines and one glutamate [25]. This is similar to the observation in Zn-specific Lbp, the zinc ion is coordinated by the side chains of three histidines and one glutamate in a slightly distorted tetrahedral geometry [28]. In the Mn-specific SBPs (PsaA and MntC), the metal is coordinated via two hisitidine residues, the third histidine is replaced by a glutamate, and the fourth position is occupied by an aspartate [23], [24]. The coordination geometry of Zn is usually tetrahedral or distorted tetrahedral (coordination number = 4) in the protein, although the coordination number can increase to five to stabilize high-energy intermediates and their flanking transition states. In contrast, the Mn^2+^ ion tends to prefer hard ligands such as the carboxylate oxygens of aspartate or glutamate, and the carboxamide oxygens of asparagine or glutamine. The nitrogen atoms of histidine imidazole groups are occasionally observed as metal ligands in Mn^2+^ metalloenzymes despite their borderline hardness, the metal coordination geometry is usually square pyramidal or trigonal bipyramidal (coordination number = 5) [13]. Accordingly, our structure indicated that Mn^2+^ is likely to be the natural ligand. However, despite great efforts, we failed to obtain any crystals of SsTroA containing Mn^2+^.

### Conformational dynamics within SBPs

Sequence comparison of SsTroA with TpTroA, *S. pneumonia* PsaA and *Synechocytis* MntC revealed amino acid sequence identities of 37%, 26% and 24%, respectively (Fig. 5). Searches with the program Dali [46] demonstrated that the closest related structures are TpTroA (PDB code 1TOA [21], rmsd 1.2 Å for 277 Cα), *Synechocytis* MntC [24] (PDB code 1XVL, rmsd 1.8 Å for 265 Cα), and *S. pneumoniae* PsaA[23] (PDB code 1PSZ ,2.0 Å for 265 Cα). Although the A-1 SBPs have a similar topological structure, subtle differences in the metal-coordinating residues are observed. For instance, His 205 in SsTroA is replaced by Glu 205 at the homologous position in PsaA and Glu 220 in MntC (Fig. 1B).

Intriguingly, in the structure of SsTroA, we observed two distinctive flexible loops mainly composed of charged residues, and these two flexible loops were designated as Loop 1 (Asn 126-Pro 38) and Loop 2 (Tyr 279-Glu 285) (Fig. 5C and D). Loop 1 is locateed between the β2-1 and β2-2 (refer to the TpTroA [21] topology nomenclature). The TpTroA and *S. pneumoniae* PsaA structures display a short loop compared to Loop 1 of SsTroA. Among the ZnuA proteins examined, this loop is longer (histidine-rich) and it is proposed that this region is possibly involved in the capture of the Zn around the metal binding site or plays an important role in the process of docking with permeases [26]. Nevertheless, the significance of this loop remains unclear because there are limited experimental results to confirm these hypotheses. Regardless, it is speculated that the mobility of this loop may possess a biological function in SsTroA [26].

It should be noted that Loop 2 is unique to SsTroA because it is absent from known SBP homologues. Moreover, the loop is surface-exposed and contains several charged residues, suggesting that it would be available to mediate higher order interactions with a ligand or protein, However, determining the significance of Loop 2 requires further structural and functional data to explain.

### NMR reveals that Zn^2+^ and Mn^2+^ are bound to nearby sites (or the same site)

**Figure 6:**
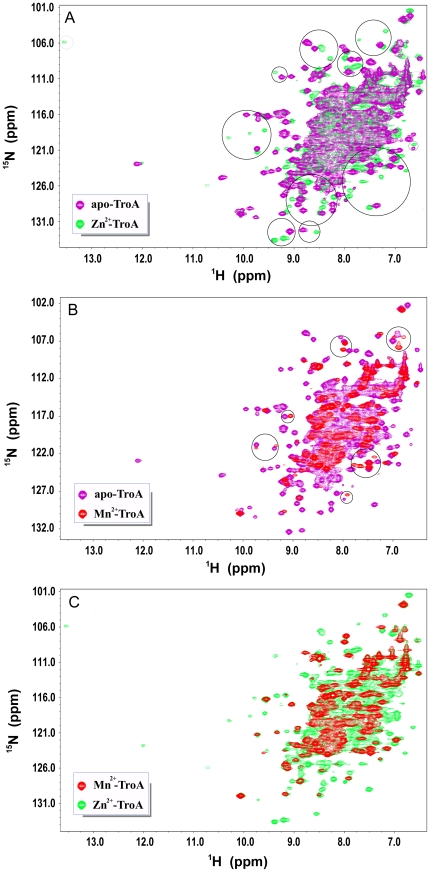
NMR spectra of SsTroA complexed with Zn^2+^ or Mn^2+^. (A) Superimposition of two-dimensional ^1^H^15^N-HSQC spectra comparing 0.5 mM apo-SsTroA (purple) with 0.5 mM Zn^2+^-SsTroA (green). (B) Overlay of 2D HSQC spectra comparing 0.5 mM apo-SsTroA (purple) with 0.5 mM Mn^2+^-SsTroA (red). (C) Comparison of 0.5 mM Zn^2+^-SsTroA (green) with 0.5 mM Mn^2+^-SsTroA (red). All spectra were acquired at 25°C in 20 mM sodium acetate (pH 6.5), and spectra were recorded at a 600-MHz ^1^H frequency. A number of the cross-peaks exhibiting significant shifts are highlighted in circles, indicating local conformational changes in the metal binding pocket.

To probe the extent of protein conformational changes in apo-SsTroA induced by Zn^2+^/Mn^2+^ binding, we recorded NMR spectroscopy at 25°C. Full ^1^H-^15^N spectra of SsTroA were acquired in the absence of metal and loaded with Zn^2+^ or Mn^2+^. A superposition of the results is presented in Figure 6. All of the cross-peaks represent backbone and side chain amide group. The spectral region encompassed is commonly referred to as the “fingerprint” for the protein conformation, and it is a known probe of minor conformational changes. As shown in Figure 6, the apoprotein (purple) maintains a similar tertiary structure compared with the metal bound proteins, suggesting that the apo-SsTroA is not randomly unfolded, but seems to fluctuate among several stable states on the intermediate time scale. Such a result is consistent with the observation of *T. pallidum*, as suggested by Lee *et al* [22].

The addition of Zn to apo-SsTroA results in substantial changes in the NMR spectrum. The spectrum of Zn^2+^-SsTroA (green) is shown in Figure 6A, displaying a large number of peaks shifts. These dramatic spectral changes indicate that the binding of Zn^2+^ induces conformational changes, demonstrating the high affinity of the apoprotein toward Zn, consistent with the ITC results described above. Also noteworthy is that many of the peaks in each protein form have similar chemical shifts, implying that the protein regions adopt similar tertiary structures. Specifically, we observed a downfield-shifted peak near 13.5 ppm in the ordered region and significant movement of multiple resonances (highlighted in circles, Fig. 6), suggesting that the involvement of the amide proton close to the Zn binding site.

In the presence of Mn^2+^, the complex solution leads to selective proton line broadening (data not shown). Two dimensional Heteronuclear Single Quantum Coherence (HSQC) NMR spectra of Mn^2+^-SsTroA are shown in Figure 6B. The number of peaks was less than the expected number of amide groups, presumably due to the paramagnetic broadening effects (Mn^2+^ is a paramagnetic agent). The cross peaks, still visible after incubating with Mn^2+^, belong to protons distal from the Mn^2+^ binding site. By contrast, the broadening and disappearance of the cross-peak strongly indicates the involvement of the amide proton close to the Mn^2+^ coordination sphere. It should be noted that almost all of the peaks in the presence of Zn^2+^ and Mn^2+^ have similar chemical shifts (Fig. 6C), indicating that the protein regions adopt identical structures when bound to Zn or Mn. This suggests that Zn^2+^ and Mn^2+^ could bind to the SsTroA in similar positions, perhaps even the same site. A similar result is observed in *N.* gonorrhoeae MntC protein, where the Zn and Mn ions compete for the same site [33].

## Discussion

ABC transport systems that contain an A-1 family SBP have emerged as key transporters that are required for virulence in several bacterial pathogens [33]. To better understand the significant role of the Tro operon for metal transition in *S. suis*, we screened the *S. suis* 2 genome [5] for orthologs of known metal-dependent enzymes and metal transporters as described previously [30] (Table S1). We identified 23 Zn-dependent proteins and three Mn-dependent proteins. Mn plays a key role in the growth of *S. suis* [47], [48], whereas *S. suis* does not encode orthologs of either Mn^2+^-specific MntH transporter or the P-type ATPase, MntA. Moreover, another putative transition metal system in *S. suis*, the Adc operon was found to exhibit homology to the *S. pneumonia* Adc operon, the latter is an ABC-type Zn permease [49]. These data imply that SsTroA maybe plays an adaptive role *in vivo*, likely modulated by SsTroR expression in response to specific environmental conditions.

The metal binding properties of TpTroA have been studied in detail [30], [31]. One puzzling observation that was unclear until now is whether or not Zn and Mn bind to the same site in TpTroA. Nuclear spin relaxation properties of resonances from protons close to the ion-binding sites can be selectively perturbed in the presence of paramagnetic ions [50]. The unpaired electrons of Mn^2+^ can interact with protons close to Mn^2+^ and the effect of the strong paramagnetic interaction of the Mn cation leads to specific changes in the corresponding NMR signals, which helps to locate the Mn^2+^ binding site [51]. Our NMR data demonstrated that Zn^2+^-SsTroA and Mn^2+^-SsTroA have very similar tertiary structures. Introduction of Mn induced the disappearance of some cross-peaks and the loss of intensity of others. Moreover, the binding of metal ions induces chemical shift changes for some of the cross-peaks (highlighted in circles, Fig. 6). However, the Zn^2+^-SsTroA and Mn^2+^-SsTroA spectra are nearly identical (except for the cross-peaks that disappear from spectrum), indicating that Zn^2+^ and Mn^2+^ share the adjacent binding region or even the same site in SsTroA.

Metal binding to the SsTroA has been proposed as a major event leading to conformational changes. From our combined NMR and ANS fluorescence data, we speculated that the metal-binding pocket of apo-SsTroA and other locally disordered folding adopts a loosely folded structure in solution. This behavior explains why metal-bound states induced the decreased ANS fluorescence intensity and changes in the NMR spectra of the metal-loaded forms. These loosely ordered structures may enhance an inducible response to the changing environmental conditions or may play a pivotal role in recognizing different divalent metal ions. In some cases, binding of a target molecule or ligand drives disordered folding to a more ordered state [52]. It should be noted that the crystallographic studies on TpTroA [21], [22] have referred to a comparatively minor folding movement upon the metal binding, and structural analysis of *Escherichia coli* ZnuA suggests that binding and release of Zn drive small yet significant conformational changes [45]. One explanation may be that the nature of protein in solution is shown to be more dynamic than suggested by crystal structures.

SsTroA presents high topological identity with lipoprotein PsaA in *S. pneumoniae*, the latter is a surface-exposed multi-functional protein detected on all known serotypes of *S. pneumoniae*. It is noteworthy that PsaA is immunogenic and being actively evaluated as a candidate vaccine component [53]. Thus far however, no data about SsTroA involvement in adhesion and/or invasion of human host tissues is yet available. There is increasing evidence that surface-related proteins are involved in bacterial pathogenesis, as exemplified by adhesion, invasion, and bacterial defense mechanisms [54]. Surface-displayed SBPs likely present an apparent dual function, metal ions transport and interaction with host cells [25]. Assessment of the potential immunogenicity of SsTroA is currently under way in our laboratory.

**Figure 7:**
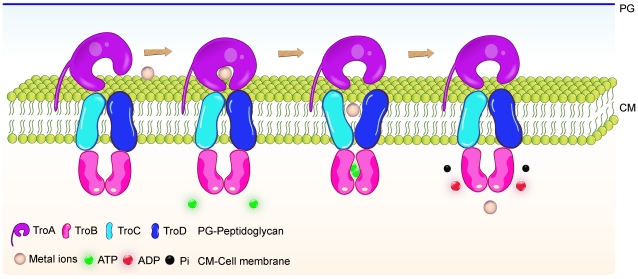
Hypothetical model of SsTroA participation in the metal ion homeostasis of *S. suis*. SsTroA is a substrate binding protein, which is anchored into the membrane via a lipid-anchor. It feeds the ligand into the translocation pathway formed by the SsTroC and SsTroD. The nucleotide binding domains (SsTroB) hydrolyze ATP to drive the transport of the ligand through the membrane.

Currently, it is premature to speculate on metal import mechanism of the SsTroABCD transporter, but it is nevertheless very tempting to hypothesize a scenario (Fig. 7). BtuF is a periplasmic binding protein (PBP) for the vitamin B12 transporter BtuCD. BtuF and TroA are classified in the same class of SBPs, which both exhibit very little domain movement upon ligand binding [20]. The complex structure of BtuCD-BtuF revealed that the conserved glutamate residues Glu 74 and Glu 202 from BtuF form salt bridges with Arg56 residues from two BtuC subunits [55], which are also found in other ABC importers [56]. Moreover, these salt bridges are critical for molecular interaction in vivo [56]. Intriguingly, we found that SsTroA also has two conserved glutamate residues Glu 107 and Glu 230 that are positioned on the surface of the N-terminal and C-terminal domains (Fig. 5A). Based on the findings described above, we predicted that SsTroA docks to the periplasmic face of SsTroCD, and charged amino acid residues probably are likely involved in salt bridge formation and contribute to the interface.

Taken together, the structural and functional data we have presented here clearly demonstrate that SsTroA binds both Zn^2+^ and Mn^2+^ with high affinity. In addition, the binding of metal ions increased the protein stability and induced conformational changes. Specifically, further analysis revealed that Zn^2+^ and Mn^2+^ bind to the SsTroA in either the same site or very nearby. The result of this work provides novel insight into the *S. suis* 2 divalent metal uptake process and establishes a framework for understanding the divalent metal homeostasis in *S. suis*. Additionally, we propose a functional model for the transport of metal ions by SsTroBCD based upon similar substrate transporters. However, further structural and functional experiments are necessary to provide evidence to support our proposed model. Studies to address these issues are underway, and will lay a solid foundation for future studies of the metal ion transport mechanism.

## Materials and Methods

### Overexpression and purification of SsTroA

The mature SsTroA domain (residues 36–317) was PCR-amplified from genomic DNA extracted from *S. suis* 05ZYH33. Primers, plasmids and bacterial strains used for gene cloning are all summarized in Table S4. The PCR product was cloned into the *Bam*H*I/Xho*I restriction sites of the pGEX-6P-1 expression vector (Amersham) to generate the pGEX-SsTroA plasmid. This construct was then transformed into *E. coli* BL21 (DE3). *E. coli* cells were grown at 37°C to mid-log-phase in LB broth supplemented with 100 mg/ml ampicillin, and then SsTroA expression was induced with 1 mM isopropyl-β-D-thiogalactopyranoside (IPTG) for 4 hours at 37°C. Cultures were harvested by centrifugation, and the cells were homogenized in PBS (140 mM NaCl, 2.7 mM KCl, 10 mM Na_2_HPO_4_, and 1.8 mM KH_2_PO_4_, pH 7.4) prior to sonication. The cell lysate was then centrifuged for 20 minutes at 35 000 g, the clarified supernatant was loaded onto a glutathione Sepharose 4B column (GE Healthcare) for 2 hours, and bound proteins were cleaved from the resin by incubating with PreScission Protease in accordance with the manufacturer's instruction for batch purification. The resulting supernatant was directly loaded onto a Superdex 200 column (GE Healthcare) equilibrated with 20 mM Tris-HCl (pH 8.0) and 50 mM NaCl. The purified protein was verified by SDS-PAGE and high purity fractions containing SsTroA were concentrated using Amicon Ultra 15 centrifugal filters (Millipore) with a 10-kDa molecular mass cut-off. Protein concentrations were determined by the BCA assay (Pierce) according to the manufacturer's protocol using bovine serum albumin as the standard. To produce uniformly ^15^N-labeled SsTroA samples for NMR studies, *E. coli* BL21 (DE3) were grown in M9 minimal medium containing 0.1% ^15^NH_4_Cl (Cambridge Isotope Laboratories) as the sole source of nitrogen. The other steps of protein expression and purification were the same as described above.

### Analytical Ultracentrifugation

A Beckman Optima XL-I analytical ultracentrifuge (Beckman Coulter Inc.) equipped with both absorbance and interference optical detection systems was used in the analytical ultracentrifugation experiment. Protein at an initial concentration of 1 mg/ml dissolved in 20 mM Tris-HCl (pH 8.0) and 50 mM NaCl was loaded into six-sector cells. Samples were centrifuged at 50,000 rpm to equilibrium at 20°C. Data were collected in continuous mode at a wavelength of 235 nm using a time interval of 300 s. The results were analyzed by the SEDFIT program (http://www.analyticalultracentrifugation.com) as described by Schuck [57].

### Preparation of apo-SsTroA

To generate the apo-SsTroA protein, SsTroA protein was treated as described previously with minor modification [31]. Briefly, the purified protein was dialyzed against 20 mM sodium acetate buffer (pH 6.5) and 20 mM EDTA for 3 days. The protein was then dialyzed against EDTA-free buffers for 3 days. All dialysis steps were performed at 4°C with stirring, and the buffer was replaced every 12 hours. Removal of metal was confirmed by ICP-MS (Inductively coupled plasma mass spectrometry). Clear plastic tips and containers were employed to prevent metal cross-contamination. All plasticwares were treated for >2 hours with 0.2 M EDTA to remove the contaminating metals, and all buffer solutions were passed through chelating Sepharose resin and stored in plastic beakers.

### Immunoblotting

The purified recombinant SsTroA proteins were screened for reactivity with antisera by western blotting. Antisera directed against recombinant SsTroA was produced in rabbits according to the standard protocol [58]. Total bacterial protein and purified protein were then resolved by 12% SDS-PAGE and transferred onto a nitrocellulose membrane (GE Healthcare) for the western blot-based detection of protein SsTroA as previously described [17]. Animal experiments were conducted in compliance with the regulations of the Beijing Administration Office of Laboratory Animal.

### Crystallization and X-Ray Data Collection

Purified protein was concentrated to 10 mg/ml in 20 mM Tris-HCl (pH 8.0) and 50 mM NaCl. Crystals with quality diffraction were obtained using the hanging drop vapor diffusion after 2 weeks at 4°C by mixing 2 µl of the protein with 2 µl of the reservoir solution containing 2.1 M ammonium sulfate and 6% v/v iso-propanol. Prior to data collection, crystals were transferred to the mother liquor containing 30% (v/v) glycerol as cryoprotectant and flash frozen in liquid nitrogen. X-ray Data were collected using a Rigaku MicroMax007 rotating-anode X-ray generator equipped with an R-AXIS IV image-plate detector. Data were processed and scaled using MOSFLM [59] and SCALA [60].

### Structure Determination and Refinement

The structure of Zn-bound SsTroA was determined using the molecular replacement method as implemented in Molrep [61] using TpTroA as the search model (PDB entry 1K0F). The structure was rebuilt and refined using COOT [62] and REFMAC5 program [63] . The crystals belong to the P4_3_ space group (a = b = 102.4Å, c = 107.3 Å, α = β = γ = 90°). The final refined structure without water molecules was used as an initial model for the complex structure. REFMAC5 [63] was again used in conjunction with COOT [62] to refine and build the complex crystal structure. The data collection and final refinement statistics are given in Table S3. Final models were validated using PROCHECK [64], and structure figures were generated by PyMOL, unless otherwise noted. Zn-SsTroA was deposited in the Protein Data Bank (PDBID: 3MFQ).

### Metal Reconstitution and Metal Content Assays

Reconstitutions with Zn^2+^ and Mn^2+^ were performed as described previously [31] with minor modifications. Briefly, 10 monomer equivalents of Zn^2+^ or Mn^2+^ were added to apo-SsTroA protein at 4°C for 12 hours. The samples, were then subjected to three rounds of concentration with Amicon Ultra 15 centrifugal filters (Millipore) followed by dilution to remove unbound metal. The metal content of recombinant SsTroA samples exchanged into metal-free 20 mM Tris-HCl (pH 8.0) and 50 mM NaCl, reconstituted SsTroA samples, and buffer controls were determined. Metal ion analysis was determined by ICP-MS, at the Analysis Center of Tsinghua University (Beijing, China). Metal content was analyzed on an XSeries II (ThermoFisher) apparatus. Each sample was quantified three times and averaged.

### Zn^2+^/Mn^2+^ Titration into apo-SsTroA by ITC

Binding of Zn^2+^ and Mn^2+^ to apo-SsTroA was determined by Isothermal Titration Calorimetry (ITC) at 25°C using a NANO ITC 2G MicroCalorimeter (TA Instruments). The metal and protein solutions were prepared in 20 mM sodium acetate buffer, pH 6.5. Prior to experiments, the reference cell was filled with deionized water, and both metal solutions and protein solutions were clarified for 10 minutes at 16,000 g and degassed for 20 minutes to eliminate air bubbles, using the ThermoVac accessory of the microcalorimeter. Upon experimental setup, the protein solution present in the sample cell was stirred at 200 rpm. After a stable baseline was achieved the titration was initiated with 25 consecutive 10 µl injections of 500 µM Zn^2+^ into the sample cell (volume = 1.5 ml) containing 90 µM apo-SsTroA,and then 200 µM Mn^2+^ was injected into 30 µM apo-SsTroA. The background heat of dilution is collected by titration with buffer alone, and the integrated data were subtracted from data obtained with apo-SsTroA present. Integrated heat data obtained for Zn^2+^ and Mn^2+^ titrations were analyzed using the NanoAnalyze software (TA Instruments) fitting them to an independent binding model. The free energy (ΔG) and entropy (ΔS) of the binding reaction can be determined with Equations 1 and 2, respectively:
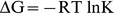
(1)

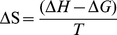
(2)


### Far-UV CD Measurements and Thermal Stability

Far-UV CD spectra were recorded at a protein concentration of 10 µM in 20 mM sodium acetate (pH 6.5) buffer on a Jasco 810 spectropolarimeter (Jasco Inc.). CD spectra were measured for apo-SsTroA alone and in the presence of 100 µM Zn^2+^ or Mn^2+^ at room temperature using a 0.1 cm quartz cell, 1 nm band width, 50 nm min^−1^ scan speed, and 1 nm step. Each curve represents the average of three accumulations and a blank containing the same buffer and detergent, buffer spectra were always subtracted, and ellipticity results were expressed in millidegree. Calculations of the fractional percentage of secondary structures were performed using the computer programs Spectra-Manager (Jasco) and K2d2 [65]. The thermal reactions were monitored on the Jasco 810 spectropolarimeter (Jasco Inc.) connected to a digitally controlled water bath (Julabo). The data were monitored for apo-SsTroA alone and loaded with Zn^2+^ or Mn^2+^ at 223 nm with a constant heating rate of 0.5°C/min,at temperatures between 25°C and 90°C. The results analysis yielded the transition temperature (Tm) as described previously [66], and the data were fit using SigmaPlot software.

### Electron Paramagnetic Resonance (EPR) Measurements

X-band EPR measurement was performed on an ER 200D SRC spectrometer (Bruker Instruments) equipped with a rectangular microwave cavity, operating at a microwave frequency of 9.53 GHz at 25°C. Three scans were collected at a microwave power of 20 mW. The center field of the EPR scans was set to 3,500 G with a sweep width of 1,000 G. EPR samples were prepared by titrating Mn^2+^ into a solution of apo-SsTroA. Stock solutions of MnCl_2_ were diluted in protein buffer with doubly distilled water. To monitor Mn^2+^ binding, one syringe was loaded with a Mn^2+^-saturated SsTroA solution (100 µM SsTroA and 100 µM MnCl_2_) in protein buffer and the other was loaded with 100 µM ZnSO_4_ freshly prepared in the same buffer. After each addition the sample was incubated for 10 minutes at 4°C prior to recording the EPR spectrum.

### Nuclear Magnetic Resonance (NMR) Spectroscopy

All NMR experiments were performed at 25°C on a Bruker DMX 600MHz spectrometer with a triple resonance cryo-probe. Uniformly ^15^N-labeled SsTroA was prepared as described above. Sample of apo-SsTroA were prepared at 0.4 mM protein concentration in 20 mM sodium acetate buffer (pH 6.5). Samples of metal bound SsTroA were prepared by the addition of 100 mM zinc acetate or manganese chloride stock to a final concentration of 1 mM metal ions in 0.4 mM protein, and the samples were then subjected to three rounds of concentration with an Amicon Ultra 15 centrifugal filters (Millipore) followed by dilution to remove unbound metal. Samples for NMR measurements contained 0.4 mM ^15^N labeled SsTroA, 95% H_2_O/5% ^2^H_2_O in 20 mM sodium acetate buffer (pH 6.5). All NMR spectra were processed and analyzed using Felix software (Accelrys Inc.).

### ANS Binding Fluorescence Assays

Fluorescence measurements were performed with a PerkinElmer LS55 fluorescence spectrometer (PerkinElmer). Emission spectra were collected at 25°C using an emission wavelength of 400–600 nm with an excitation wavelength of 380 nm [66]. Apo-protein (5 µM) was mixed with 500 µM ANS in 20 mM sodium acetate buffer (pH 6.5). Zn^2+^ and Mn^2+^ were added to the mixture of 5 µM apo-SsTroA and 500 µM ANS to 10 µM final concentration, and the mixtures were incubated at 25°C for 15 minutes prior to collecting the excitation spectrum.

## Supporting Information

Figure S1 **Biochemical characterization of recombinant SsTroA protein.** (A) 12**%** SDS-PAGE analysis of over-expressed SsTroA protein. Lane 1, soluble protein; Lane 2, inclusion body; Lane 3, whole cell; Lane 4, Non-induced bacteria; Lane 5, samples treated by prescission protease; Lane 6, purified SsTroA; M, Molecular weight markers as indicated. (B) Western blotting analysis of SsTroA. Lane 1, purified recombinant SsTroA; Lane 2, negative control (the unrelated protein sample, Ss1661 protein); Lane 3, *S. suis* whole cell lysates.
(TIF)

Figure S2 **The oligomeric state of the recombinant SsTroA.** (A) The size-exclusion gel-filtration chromatography profiles of SsTroA and SDS-PAGE (left inset). Two peaks from the gel-filtration show the same SsTroA protein on the SDS-PAGE. (B) Sedimentation velocity analysis of SsTroA. The profile shows the calculated molar mass distribution of SsTroA.
(TIF)

Table S1 **Genome-wide search for genes with zinc- and manganese-involvement.**
(DOC)

Table S2 **Thermodynamics of binding of Zn^2+^/Mn^2+^ to SsTroA measured by ITC at 25°C.**
(DOC)

Table S3 **Statistics of X-ray diffraction data and refinement details.**
(DOC)

Table S4 **Primers, plasmids and bacterial strains for molecular cloning.**
(DOC)
