# Supplementary figures and images for: Insight into the Interaction of Metal Ions with TroA from Streptococcus suis
Source: PLoS One. 2011 May 18;6(5):e19510. doi: 10.1371/journal.pone.0019510 (PMC3097204; doi:10.1371/journal.pone.0019510)

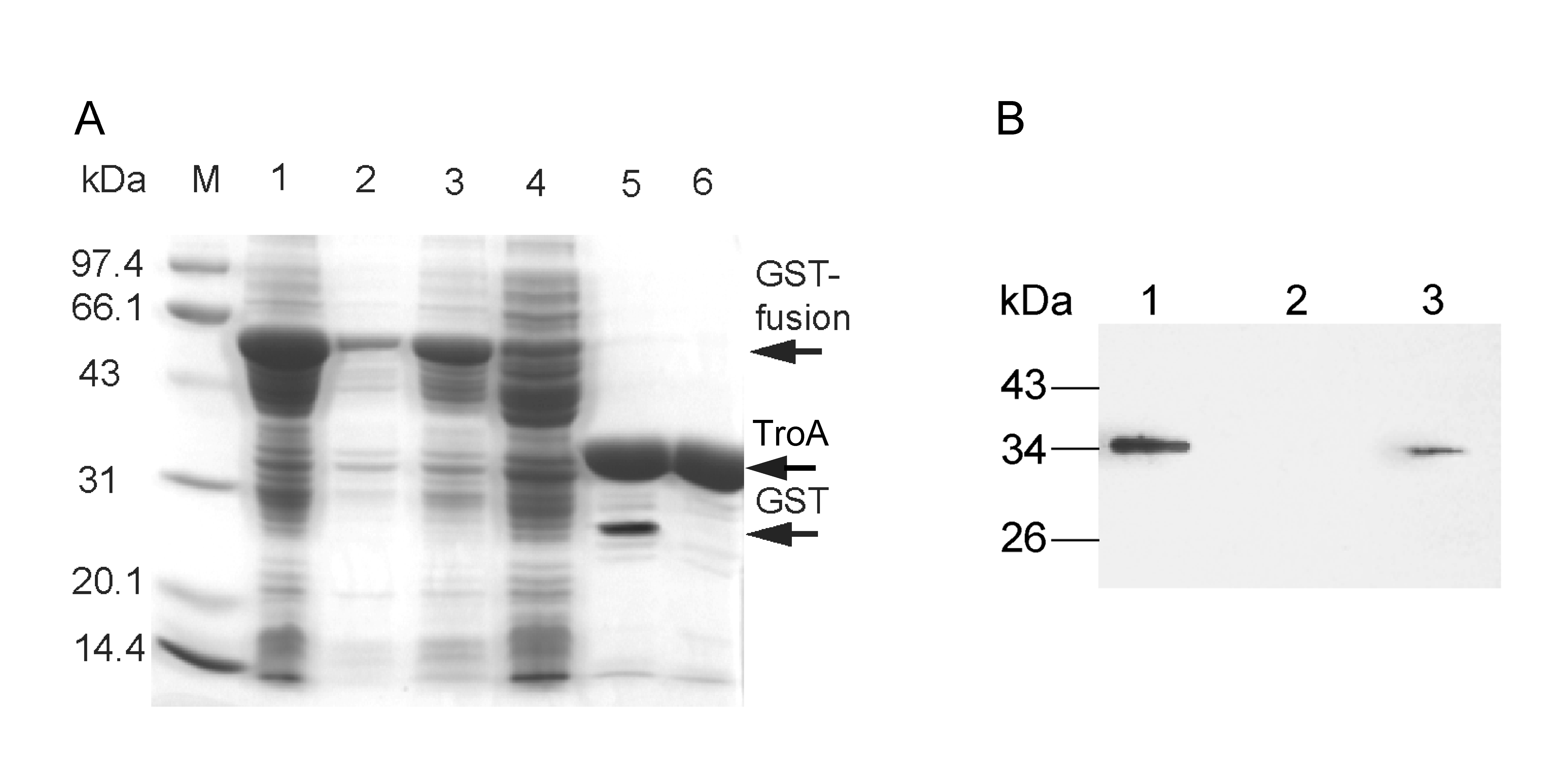

Supplement: Figure S1 — Biochemical characterization of recombinant SsTroA protein. (A) 12% SDS-PAGE analysis of over-expressed SsTroA protein. Lane 1, soluble protein; Lane 2, inclusion body; Lane 3, whole cell; Lane 4, Non-induced bacteria; Lane 5, samples treated by prescission protease; Lane 6, purified SsTroA; M, Molecular weight markers as indicated. (B) Western blotting analysis of SsTroA. Lane 1, purified recombinant SsTroA; Lane 2, negative control (the unrelated protein sample, Ss1661 protein); Lane 3, S. suis whole cell lysates. (TIF) [file pone.0019510.s001.tif]

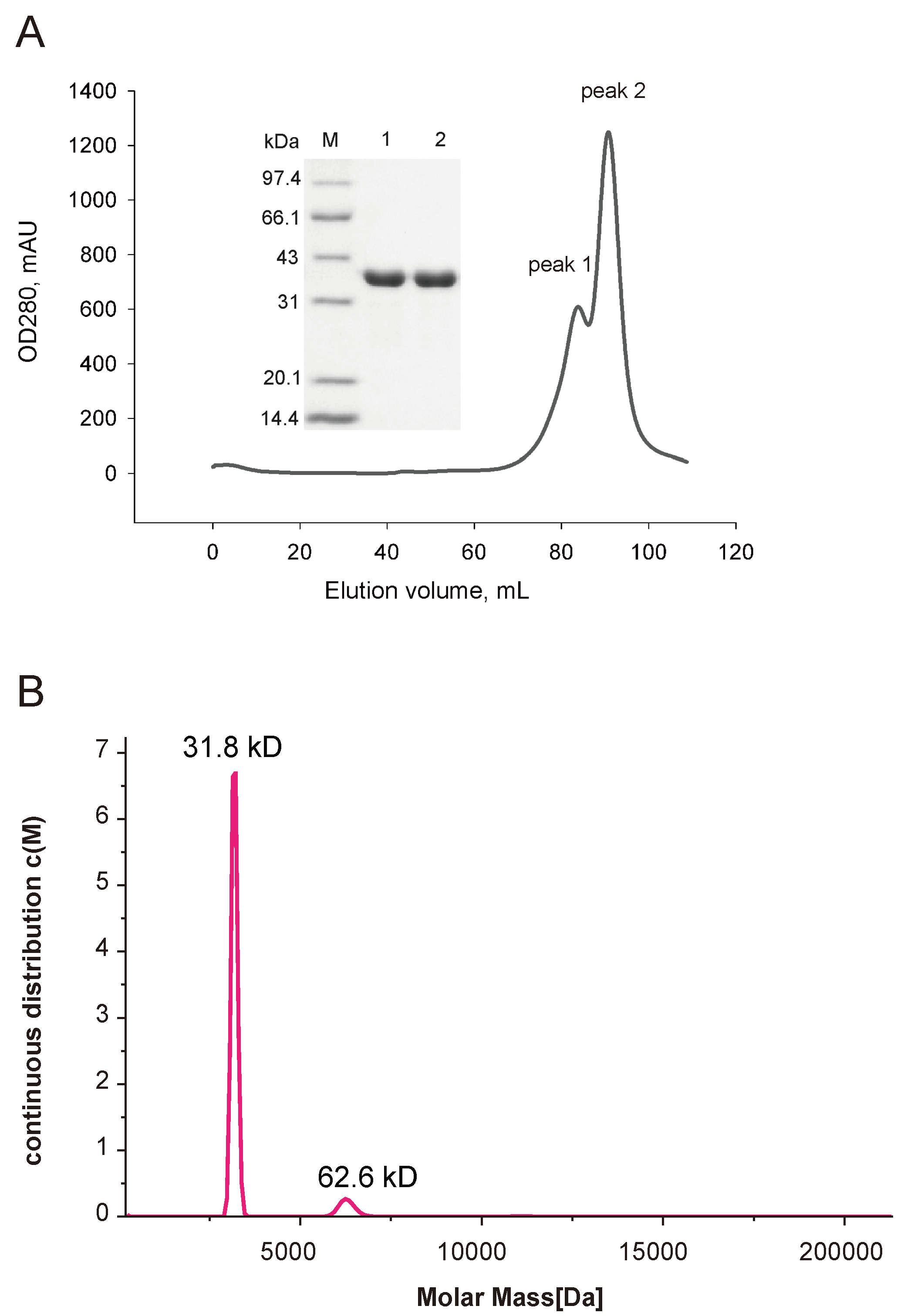

Supplement: Figure S2 — The oligomeric state of the recombinant SsTroA. (A) The size-exclusion gel-filtration chromatography profiles of SsTroA and SDS-PAGE (left inset). Two peaks from the gel-filtration show the same SsTroA protein on the SDS-PAGE. (B) Sedimentation velocity analysis of SsTroA. The profile shows the calculated molar mass distribution of SsTroA. (TIF) [file pone.0019510.s002.tif]
