# Supplementary material for: Insight into the Interaction of Metal Ions with TroA from Streptococcus suis
Source: PLoS One. 2011 May 18;6(5):e19510. doi: 10.1371/journal.pone.0019510 (PMC3097204; doi:10.1371/journal.pone.0019510)
Supplement: Table S1 — Genome-wide search for genes with zinc- and manganese-involvement. (DOC) [file pone.0019510.s003.doc]

**Table S1** Genome-wide search for genes with zinc- and manganese-involvement

| Functional annotation | Gene code |
| --- | --- |
| Orthologs of transition metal-dependent enzymes |  |
| Zinc |  |
| Neutral zinc metallopeptidases | 05SSU154 |
| Zinc-containing alcohol dehydrogenase superfamily | 05SSU279 |
|  | 05SSU319 |
| Periplasmic solute binding protein | 05SSU331 |
| Ketose-bisphosphate aldolase | 05SSU336 |
|  | 05SSU338 |
|  | 05SSU339 |
| Isoleucyl-tRNA synthetase | 05SSU489 |
| Oligoendopeptidase F | 05SSU728 |
| ATP-dependent protease Clp | 05SSU837 |
| Zinc metalloprotease zmpC precursor | 05SSU1022 |
| Dihydrofolate reductase | 05SSU1077 |
| Cytidine deaminase | 05SSU1084 |
| Zn-dependent dehydrogenases | 05SSU1389 |
| Formamidopyrimidine-DNA glycosylase | 05SSU1396 |
| Zn-dependent protease | 05SSU1478 |
| Mannose-6-phosphate isomerase | 05SSU1607 |
| Cytidine/deoxycytidylate deaminase | 05SSU1622 |
| Metalloprotease | 05SSU1657 |
| Membrane-associated Zn-dependent proteases 1 | 05SSU1962 |
| Adenosine deaminases | 05SSU2053 |
| Metalloendopeptidase | 05SSU2082 |
| Zn-dependent peptidases | 05SSU2079 |
|  | 05SSU2080 |
| Manganese |  |
| Manganese-dependent superoxide dismutase | 05SSU1539 |
| Manganese-dependent inorganic pyrophosphatase | 05SSU1665 |
| Mannonate Dehydratase | 05SSU1157 |
| Orthologs of metal transporters |  |
| AdcABCR | 05SSU109-05SSU112 |
| TroABCDR | 05SSU2083-05SSU2087 |
| Cobalt ABC transporter permease | 05SSU740-05SSU742 |
| Cadmium-transporting ATPase | 05SSU309 |
| Potassium uptake protein, TrkH | 05SSU1759 |
| Potassium uptake protein, TrkA | 05SSU1760 |
| Mg^2+^ and Co^2+^ transporters | 05SSU1829-05SSU1830 |
| Copper-transporting P-type ATPase | 05SSU220-05SSU222 |
|  | 05SSU13840-5SSU1386 |
| Zinc transporter ZIP | 05SSU1302 |
