# Supplementary material for: Insight into the Interaction of Metal Ions with TroA from Streptococcus suis
Source: PLoS One. 2011 May 18;6(5):e19510. doi: 10.1371/journal.pone.0019510 (PMC3097204; doi:10.1371/journal.pone.0019510)
Supplement: Table S2 — Thermodynamics of binding of Zn2+/Mn2+ to SsTroA measured by ITC at 25°C. (DOC) [file pone.0019510.s004.doc]

**Table S2.** Binding thermodynamics of Zn^2+^/Mn^2+^ to SsTroA measured by ITC at 25 ℃

|  | Ka | ΔG | ΔH | -TΔS | ΔS | n |
| --- | --- | --- | --- | --- | --- | --- |
| Metal ions | ×10^7^ M^-1^ | kJ/mol | kJ/mol | kJ/mol | J K^-1^ mol^-1^ |  |
| Zn^2+^ | 3.96 | -37.6 | -24.94 | 12.66 | -42.65 | 0.92 |
| Mn^2+^ | 2.2 | -36.2 | -29.53 | 6.67 | -22.4 | 0.97 |
