# Supplementary material for: Insight into the Interaction of Metal Ions with TroA from Streptococcus suis
Source: PLoS One. 2011 May 18;6(5):e19510. doi: 10.1371/journal.pone.0019510 (PMC3097204; doi:10.1371/journal.pone.0019510)
Supplement: Table S3 — Statistics of X-ray diffraction data and refinement details. (DOC) [file pone.0019510.s005.doc]

**Table S3.** Statistics of X-ray diffraction data and refinement details

| **Data collection** |  |
| --- | --- |
| Space group | *P*4_3_ |
| Unit cell parameters | a=b=102.36Å, c=107.33 Å, α=β=γ=90° |
| Number of reflections | 195877 |
| Completeness (%) | 96.8 (99.9) |
| *R*merge (%) | 9.5 (49.1) |
| *I/*σ (*I*) | 14.965 (4.565) |
| **Refinement** |  |
| Resolution | 50-2.6 |
| R*work* (%) | 0.226 |
| R*free* (%) | 0.256 |
| RMS bonds (Å) | 0.003 |
| RMS angle (deg) | 0.61 |
| Bound ligand | Zn |

Values in parentheses are for the shell of highest resolution
