# Supplementary material for: Insight into the Interaction of Metal Ions with TroA from Streptococcus suis
Source: PLoS One. 2011 May 18;6(5):e19510. doi: 10.1371/journal.pone.0019510 (PMC3097204; doi:10.1371/journal.pone.0019510)
Supplement: Table S4 — Primers, plasmids and bacterial strains for molecular cloning. (DOC) [file pone.0019510.s006.doc]

**Table S4.** Primers, plasmids and bacterial strains for molecular cloning

|  | Characteristics or function | Source |
| --- | --- | --- |
| Primers |  |  |
| TroA-for | 5’-CGGGATCCACAGAAGGCTCTAGTAGC-3’ | This work |
| TroA-rev | 5’-CCGCTCGAGTTATTTCAGATATTTAAC-3’ | This work |
| Plasmids |  |  |
| pGEM-T vector | Amp^r^; T vector | Promega |
| pGEX-6P-1 | Amp^r^; recombinant expression plasmid | Amersham |
| Strains |  |  |
| DH5α | Genetically modified *E. coli,* cloning host | Qiagen |
| BL21(DE3) | Genetically modified *E. coli,* expression host | Novagen |
